# Surveillance recommendations for *DICER1* pathogenic variant carriers: a report from the SIOPE Host Genome Working Group and CanGene-CanVar Clinical Guideline Working Group

**DOI:** 10.1007/s10689-021-00264-y

**Published:** 2021-06-25

**Authors:** Jette J. Bakhuizen, Helen Hanson, Karin van der Tuin, Fiona Lalloo, Marc Tischkowitz, Karin Wadt, Marjolijn C. J. Jongmans, SIOPE Host Genome Working Group, Beate B. Dörgeloh, Roula A. Farah, Stavros Glentis, Lisa Golmard, Juliane Hoyer, Kirsi Jahnukainen, Rosalyn Jewell, Axel Karow, Katharina Katsibardi, Michaela Kuhlen, Andrea Meinhardt, Karolina Nemes, Anna Poluha, Tim Ripperger, Nicolas Waespe, CanGene-CanVar Clinical Guideline Working Group, Julian Adlard, Munaza Ahmed, Bernadette Brennan, Tabib Dabir, D. Gareth Evans, Anna Kelsey, Kelly Kohut, Anju Kulkarni, Alex Murray, Kai Ren Ong, Anthony Penn, Thomas Semple, Emma R. Woodward, Expert Network Members, Rachel S. van Leeuwaarde, Annemieke S. Littooij, Johannes H. M. Merks, Åse K. Rasmussen, Hanneke M. van Santen, Stephanie E. Smetsers

**Affiliations:** 1 Princess Máxima Center for Pediatric Oncology, Utrecht, The Netherlands; 2 Department of Genetics, University Medical Center Utrecht, PO Box 85090, 3508 AB Utrecht, The Netherlands; 3 Department of Clinical Genetics, St George’s University Hospitals NHS Foundation Trust, London, UK; 4 Department of Clinical Genetics, Leiden University Medical Center, Leiden, The Netherlands; 5 Manchester Centre for Genomic Medicine, Manchester University NHS Foundation Trust, Manchester, UK; 6 Department of Medical Genetics, National Institute for Health Research Cambridge Biomedical Research Centre, Cambridge University Hospital NHS Foundation Trust, Cambridge, UK; 7 Department of Clinical Genetics, Copenhagen University Hospital Righospitalet, Copenhagen, Denmark

**Keywords:** *DICER1*, Surveillance, Hereditary, Cancer predisposition syndrome

## Abstract

DICER1 syndrome is a rare genetic disorder that predisposes to a wide spectrum of tumors. Developing surveillance protocols for this syndrome is challenging because uncertainty exists about the clinical efficacy of surveillance, and appraisal of potential benefits and harms vary. In addition, there is increasing evidence that germline *DICER1* pathogenic variants are associated with lower penetrance for cancer than previously assumed. To address these issues and to harmonize DICER1 syndrome surveillance programs within Europe, the Host Genome Working Group of the European branch of the International Society of Pediatric Oncology (SIOPE HGWG) and Clinical Guideline Working Group of the CanGene-CanVar project in the United Kingdom reviewed current surveillance strategies and evaluated additional relevant literature. Consensus was achieved for a new surveillance protocol and information leaflet that informs patients about potential symptoms of *DICER1*-associated tumors. The surveillance protocol comprises a minimum program and an extended version for consideration. The key recommendations of the minimum program are: annual clinical examination from birth to age 20 years, six-monthly chest X-ray and renal ultrasound from birth to age 6 years, and thyroid ultrasound every 3 years from age 8 to age 40 years. The surveillance program for consideration comprises additional surveillance procedures, and recommendations for *DICER1* pathogenic variant carriers outside the ages of the surveillance interval. Patients have to be supported in choosing the surveillance program that best meets their needs. Prospective evaluation of the efficacy and patient perspectives of proposed surveillance recommendations is required to expand the evidence base for DICER1 surveillance protocols.

**Supplementary Information:**

The online version contains supplementary material available at 10.1007/s10689-021-00264-y.

## Introduction

DICER1 syndrome is an autosomal dominant hereditary tumor predisposition syndrome that predisposes individuals to a variety of tumors, both benign and malignant [1, 2]. In 2009, disease-associated variants in the *DICER1* gene were first described in families with multiple cases of pleuropulmonary blastoma (PPB) [2]. Over time, numerous other manifestations have been associated with pathogenic germline variants in *DICER1*, including lung cysts, multinodular goiter, thyroid cancer, ovarian sex-cord stromal tumors, and cystic nephroma [1, 3–5]. Less commonly described manifestations in individuals with DICER1 syndrome include nasal chondromesenchymal hamartoma (NCMH), ciliary body medulloepithelioma (CBME), Wilms tumors, primary brain tumors, mesenchymal hamartoma of the liver, and sarcomas of various sites [1, 6–11]. The majority of tumors occur in infancy, childhood, and adolescence [12]. Macrocephaly is one of the few non-neoplastic features of DICER1 syndrome, which may also include retinal and structural renal abnormalities [10, 13, 14]. Possibly additional tumors or non-neoplastic features will be linked to DICER1 syndrome in the future.

In 2018, two independent groups proposed DICER1 syndrome surveillance protocols [15, 16]. Developing surveillance protocols for DICER1 syndrome is challenging, not least due to uncertainty about the efficacy of surveillance for individuals with germline pathogenic *DICER1* variants. Proposed surveillance protocols aim to reduce *DICER1*-associated morbidity and mortality through early detection of tumors by imaging of several organs. However, the clinical utility of these protocols remains to be validated [15–18]. The natural history (i.e., rate of malignant transformation) and growth rate of most *DICER1-*associated tumors has not yet been investigated [1, 19]. Another challenge in developing surveillance protocols is the potential harm associated with surveillance. Potential harms in DICER1 syndrome surveillance protocols include overtreatment (e.g., unnecessary surgery for asymptomatic benign cysts detected on surveillance), need for sedation in young children during imaging procedures, radiation exposure and psychosocial burden of repeated investigations and false-positive findings [20]. Given these potential harms, the question has been raised whether less invasive and less frequent surveillance regimes are reasonable. This issue has grown in importance in light of two recent findings. Firstly, approximately 95% of non-index case individuals with germline pathogenic *DICER1* variants did not develop a tumor by age 10 years [12]. Secondly, germline *DICER1* pathogenic variants may be more common in the general population than previously thought, reflecting a lower penetrance than previously assumed [19]. The current incidence of loss-of-function (LOF) variants in the gnomAD data set (71,702 genomes, accessed 23/10/20) is 1:5121 [21].

To address these issues, the Host Genome Working Group of the European branch of the International Society of Pediatric Oncology (SIOPE HGWG) organized a meeting during which current surveillance protocols for DICER1 syndrome were reviewed and new surveillance recommendations were proposed. In addition, the Clinical Guideline Working Group of the CanGene-CanVar project in the United Kingdom was invited as a collaborator in the guideline development process to harmonize surveillance programs within Europe. The joint recommendations, which both overlap and incorporate modifications compared to previous protocols, are presented and explained in this report.

## Methods

In January 2020, the SIOPE HGWG met in Hannover, Germany, to reassess current surveillance strategies for *DICER1* pathogenic variant carriers. Eighteen professionals from ten countries were present, including clinical geneticists and pediatric oncologists.

Prior to this meeting, a literature review was performed to identify articles on surveillance in DICER1 syndrome. The DICER1 syndrome surveillance protocol proposed by Schultz and colleagues was selected as key publication for discussion [16]. SIOPE HGWG members were surveyed for their opinions on these surveillance recommendations and were asked about their current practice and experiences. Surveillance recommendations were draft based on the survey responses and expert discussions during the SIOPE HGWG meeting.

Following this meeting, experts in specific fields (e.g., endocrinology, pathology, radiology, and gynecology) were consulted for their input and the literature search was extended to identify additional relevant articles. A PubMed search was conducted using the keywords “DICER1” [title/abstract] and “humans”[MeSH Terms] in combination with the keywords “screening” [title/abstract] OR “surveillance” [title/abstract] OR “incidence”[title/abstract] OR “penetrance”[title/abstract] OR synonyms for the various DICER1-associated tumors (Supplemental Table 1). Articles written in English were included and there was no data limit. In total, 225 articles were found. Articles were included if they contained any data on the incidence, penetrance, or surveillance of *DICER1*-associated tumors. In addition, the CanGene-CanVar group in the United Kingdom was invited as a partner in the guideline development process. Similar to the SIOPE HGWG, the CanGene-CanVar Clinical Guideline Working Group aimed to develop new DICER1 syndrome surveillance protocols. To harmonize surveillance programs within Europe, it was appropriate to write joint guidance. Revised protocols were assessed by all participants of the SIOPE HGWG meeting and members of the CanGene-CanVar group to ensure consensus. In addition, patient representatives (n = 7) from the UK, Denmark, Switzerland, Germany, and the Netherlands were consulted for their opinion on the comprehensibility of the information leaflet.

For evidence grading we used a scale that was introduced in the ERN GENTURIS Cancer Surveillance Guideline for individuals with PTEN hamartoma tumor syndrome: (i) strong evidence: consistent evidence and new evidence unlikely to change recommendation and expert consensus; (ii) moderate evidence: expert consensus or majority decision but with inconsistent evidence or significant new evidence expected and (iii) weak evidence: inconsistent evidence and limited expert agreement [22].

## Results

**Fig. 1:**
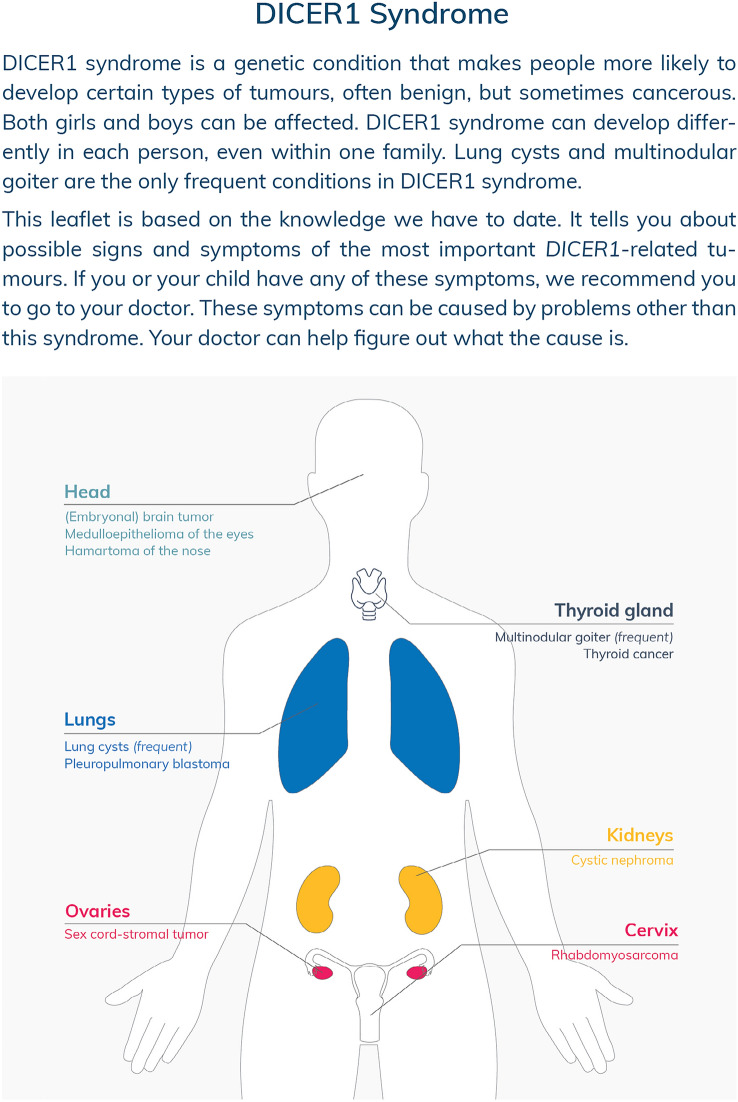

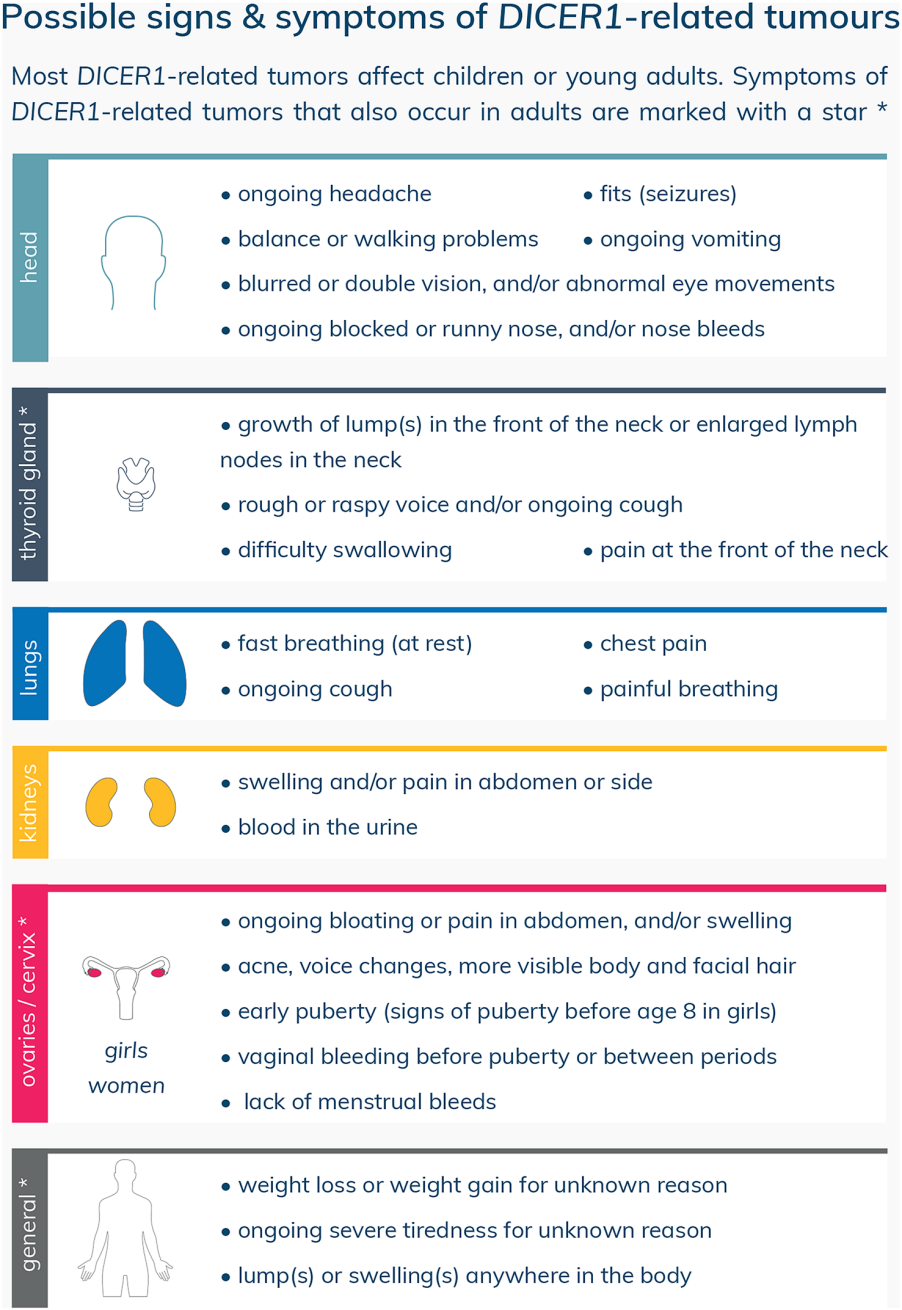
DICER1 syndrome patient information leaflet: possible signs and symptoms of *DICER1*-associated tumors

The SIOPE HGWG and CanGene-CanVar propose new surveillance recommendations for *DICER1* pathogenic variant carriers, summarized in a surveillance protocol (Table 1) and a patient information leaflet (Fig. 1). The aim of the information leaflet is to inform patients, parents/guardians, and general practitioners about possible signs and symptoms of *DICER1*-associated tumors in order to reduce diagnostic delay. This leaflet also comprises rare *DICER1*-associated tumors, like nasal chondromesenchymal hamartoma (NCMH) and primary brain tumors, for which we do not recommend standardized surveillance.

**Table 1:** SIOPE HGWG and CanGene-CanVar surveillance protocol for *DICER1* pathogenic variant carriers

	Minimum program*	Extended program for consideration**
General surveillance	Annual clinic review for symptoms and clinical examination where appropriate from birth to age 20 years	
Pulmonary surveillance	US 3^rd^ trimester of pregnancy^a^ Six-monthly chest X-ray from birth to age 6 years	Low-dose chest CT in first year and at age 2.5–3 years Single chest X-ray at the time of diagnosis if DICER1 syndrome is diagnosed after age 6 years
Renal surveillance	Six-monthly abdominal US from birth to age 6 years	Single abdominal US at the time of diagnosis if DICER1 syndrome is diagnosed after age 6 years
Thyroid surveillance	Thyroid US every 3 years from age 8 to age 40 years Annual neck palpation from age 8 to age 20 years^**^ Thyroid function monitoring during pregnancy^b^	Thyroid US at the time of diagnosis if DICER1 syndrome is diagnosed between age 40 and age 50 years
Surveillance of the ovaries		Annual US of the ovaries from age 8 to age 40 years^c^

*SIOPE HGWG* Host Genome Working Group of the European branch of the International Society of Pediatric Oncology, *US* ultrasound, *CT* computed tomography

^a^For pregnant women whose fetuses are prenatally diagnosed with a pathogenic germline *DICER1* variant or are at risk to be affected (i.e., 50% chance of inheriting a germline *DICER1* pathogenic variant from either the maternal or paternal side)

^b^For pregnant women with a pathogenic germline *DICER1* variant

^c^Transition to transvaginal ultrasound should be considered in older adolescents and adults when the ovaries are not visible with transabdominal ultrasound

*Based on expert consensus or majority decision but with inconsistent evidence or significant new evidence expected, except for annual neck palpation which is based on inconsistent evidence and limited expert agreement

**Based on inconsistent evidence and limited expert agreement

The surveillance protocol comprises a minimum program as well as an extended version for consideration. The minimum program contains surveillance procedures (i.e., clinical examination and imaging-based surveillance) for which the SIOPE HGWG and CanGene-CanVar assume that the benefits of surveillance outweigh the potential harms. The minimum program recommendations are based on expert consensus or majority decision but with inconsistent evidence or significant new evidence expected, except for annual neck palpation which is based on inconsistent evidence and limited expert agreement. We recommend annual clinical review from birth to age 20 for possible signs and symptoms of *DICER1*-associated tumors (described in the information leaflet) and continued education and updates on proper vigilance. Annual physical examination should preferably be performed by a ‘key’ clinician who is also responsible for arranging and reviewing imaging-based surveillance to ensure appropriate follow-up. We suggest that surveillance is provided and coordinated by pediatric oncologists in *DICER1* pathogenic variant carriers from birth to age 8 years, because of the broad spectrum of childhood tumors that can occur in this time period. Patients older than age 8 years can be seen by (pediatric) endocrinologists since the minimum surveillance program from this age onwards comprises only thyroid surveillance. Given the rarity of DICER1 syndrome, surveillance should preferably be performed in a center with expertise. If this is not possible, we strongly advise clinicians to consult subspecialists with expertise in managing patients with DICER1 syndrome in case of suspicious physical examination or imaging findings. *DICER1* pathogenic variant carriers who have experienced a tumor may require additional surveillance of the organ that was affected. This needs to be discussed within a multidisciplinary team.

The upper and lower age limits for surveillance are based on the typical age of onset of *DICER1*-associated tumors, covering approximately 90–95% of reported cases [1, 3, 5, 12, 17, 23, 24], in accordance with practice regarding other cancer predisposition syndromes, e.g. *BRCA*1/*BRCA2*-associated familial breast and ovarian cancer [25]. The extended program for consideration comprises additional surveillance procedures and recommendations for patients diagnosed with DICER1 syndrome outside the ages of the surveillance interval. These recommendations are based on inconsistent evidence and limited expert agreement. The extended program promotes personalized surveillance and shared decision making. How patients (or parents) perceive benefits and risks of surveillance can differ greatly between individuals [26–29]. Therefore, we encourage clinicians to thoroughly discuss potential benefits and risks with patients (and/or parents). Patients have to be supported in choosing the surveillance program that best meets their needs.

## Rationale for the recommendations

### Pulmonary surveillance

PPB is one of the most important causes of *DICER1*-associated morbidity and mortality [1, 12]. Clinically relevant PPBs are categorized into type I, II, or III. Type I PPB can progress to the more aggressive type II or III PPB if left untreated. The 5-year disease-free survival of type I PPB is significantly better than for type II and III PPB (79%, 59%, and 37%, respectively) [23]. These findings support the clinical utility of pulmonary imaging surveillance in *DICER1* pathogenic variant carriers. It is important to note that type I PPB can also spontaneously regress to type Ir (regressed) PPB, which is identified with a substantial frequency among adults with DICER1 syndrome and harbor little-to-no malignant potential, although, at present, there are no data regarding the proportion of progressing and regressing asymptomatic lung cysts [12].

Regular imaging with chest X-rays, followed by chest CT in cases with suspicious abnormalities on X-ray, is currently assumed to be the optimal surveillance method [30]. This approach balances the risk of radiation and possible need for sedation, against the risk of undetected disease. The classification of lung cysts on imaging determines whether subsequent follow-up by chest CT and/or surgery is needed [17]. Concerning features suggestive of PPB include pneumothorax, the presence of more than one separate cystic pulmonary lesion, large unilocular cysts of over 2 cm diameter, thick or irregularly septated cysts, pulmonary cysts not detected on antenatal ultrasound (c.f. cystic congenital pulmonary airway malformation [CPAM]), and the presence of pleural effusion.

The SIOPE HGWG and CanGene-CanVar agree on currently used pulmonary surveillance imaging modalities, but advocate for modifying the age at which surveillance should stop. Current DICER1 syndrome surveillance protocols recommend chest X-rays every four to six months from birth to age 8 years, followed by annual chest X-rays until age 12 or age 18 years [15, 16]. Although type Ir PPB can present in individuals of any age, clinically significant PPB typically presents in infants and children [1, 12, 23]. The International Pleuropulmonary Blastoma registry reported clinical data from 350 PPB Cases [23]. Twenty-five percent of these cases (n = 89) were type I PPB, 7% (n = 26) were type Ir, and 67% (n = 235) were type II or III PPB. The age by which 95% of the cases of type I, type II, and type III or type II-III PPB were diagnosed was 2.5 years, 6.8 years and 5.3 years, respectively. These findings demonstrate that almost all clinically relevant PPB cases present within the first six years of life. Accordingly, pulmonary surveillance at ages older than age 6 years, will provide marginal extra benefit, while the potential harm of overdiagnosis and excessive medicalization increases. We therefore recommend six-monthly chest X-ray from birth to age 6 years. A single, baseline chest X-ray at the time of diagnosis can be considered in individuals diagnosed after age 6 years.

Another surveillance option for consideration is a low-dose chest CT (without contrast) in the first year and at the age of 2.5–3 years, prior to the peak incidence of type II and III PPB [23]. This is in agreement with current surveillance protocols for *DICER1* pathogenic variant carriers [15, 16]. The sensitivity of chest CT for detection of small pleuropulmonary blastoma cysts is superior to that of chest X-ray [30]. This potential benefit should, however, be carefully balanced against the potential risk of false-positive findings that may lead to unnecessary interventions, and against the risk of inducing cancer by ionizing radiation exposure, particularly in children. Developing organs and tissues are more sensitive to the carcinogenic effects of ionizing radiation and the longer post-exposure life expectancy in children increases the lifetime risk of developing radiation-induced cancer [31]. A further potential burden of CT scans in young children is the requirement for general anesthesia, although new CT scan techniques (e.g., ultrafast free breathing chest CT without anesthesia) and involvement of play-therapists may eliminate this burden [32]. Ongoing improvements on Magnetic Resonance Imaging (MRI) will potentially result in limited ionizing radiation exposure during DICER1 syndrome surveillance in the future.

### Renal surveillance

Renal tumors reported in individuals with DICER1 syndrome include cystic nephroma (CN), Wilms tumor and anaplastic sarcoma [1]. CN is one of the most common *DICER1*-associated tumors (estimated cumulative incidence among carriers of a germline *DICER1* pathogenic variant [index and non-index case patients] is ~ 7% by the age of 6 years), whereas malignant diseases as Wilms tumors and anaplastic sarcoma are very rare [12]. It has been suggested that renal anaplastic sarcoma evolves from CN [33, 34]. Earlier diagnosis of CN may result in less extensive surgery and may prevent progression to renal anaplastic sarcoma.

To enable early detection of renal tumors, the SIOPE HGWG and CanGene-CanVar recommend six-monthly abdominal ultrasound from birth to 6 years of age. We propose stopping renal surveillance at a younger age than advised in current DICER1 syndrome surveillance protocols [15, 16]. Given the rarity of Wilms tumors in *DICER1* pathogenic variant carriers, we adjusted the age and interval at which renal surveillance should be performed to the available data for CN which primarily affects children younger than 5 years of age [1, 12, 17].

Consideration should be given to a single, baseline abdominal ultrasound in individuals who are diagnosed with a pathogenic germline variant in *DICER1* after the age of 6. Even though the risks of developing a *DICER*-associated renal tumor after age 6 years is very low, two children with DICER1 syndrome were reported with CN after the age of 10 years in the literature [12].

### Thyroid surveillance

Multinodular goiter (MNG) is the most common DICER1 syndrome associated condition, especially in women. *DICER1*-associated MNG is usually diagnosed in the first decades of life [1]. Khan and colleagues calculated the cumulative incidence of either MNG diagnosis or thyroidectomy (for MNG or thyroid nodules) in a family-based cohort study including 145 individuals with a germline *DICER1* pathogenic variant and 135 family controls [5]. The estimated cumulative incidence of MNG or thyroidectomy by the age of 40 years was 75% in women (95% CI 59–89) and 17% in men (95% CI 7.3–35) with DICER1 syndrome. Thyroid disease can newly arise or pre-existing goiter can worsen during or shortly after pregnancy [35].

Thyroid cancer, especially differentiated thyroid cancer (DTC), has also been described in individuals with DICER1 syndrome, but the cumulative incidence is unknown [5, 12, 24, 36]. Khan and colleagues calculated that carriers of a germline *DICER1* pathogenic variant have a 16-fold increased risk of developing DTC (95% CI 4.3–41) compared with the expected frequencies from the Surveillance, Epidemiology and End Results (SEER) Program [5]. To date, approximately 30 patients with (suspected) *DICER1*-associated thyroid cancer have been reported [24]. More than 90% of these patients were diagnosed with thyroid cancer before the age of 40 years, with a peak between age 10 and 20 years. The risk of DTC in carriers of a germline *DICER1* pathogenic variant might be secondary to the greatly increased prevalence of benign thyroid nodules, although, given the low number of reported patients with *DICER1-*associated thyroid cancer, it has been suggested that only a small percentage of benign thyroid nodules progress to thyroid cancer [5, 12, 24].

Neck palpation in combination with thyroid ultrasound has been recommended as surveillance modalities for *DICER1* pathogenic variant carriers [15, 16, 37]. The sensitivity to detect thyroid cancer is higher for ultrasound than for neck palpation, but this comes at the expense of lower specificity [38]. Even though no studies directly examined the harms associated with thyroid ultrasounds in individuals with DICER1 syndrome, overdiagnosis and overtreatment is a concern. Since DICER1 syndrome is characterized by a high prevalence of benign nodules [5], interpretation of thyroid ultrasound findings is challenging. The Thyroid Imaging Reporting and Data Systems created by the American College of Radiology (ACR-TIRADS) and European Thyroid Association (EU-TIRADS) have not been validated in specific populations with a high prevalence of benign nodular thyroid disease, like individuals with DICER1 syndrome. Therefore, no recommendations for using this reporting system can be made. Findings from a retrospective evaluation of clinical, molecular and histological data of ten patients with DICER1-associated DTCs, raises concern of unnecessary exposure of radioiodine treatment given the tumors’ low propensity for metastasis [24].

The great majority of *DICER1*-associated thyroid tumors reported to date behaved in an indolent manner [24]. Exceptions are clinically aggressive *DICER1*-mutated anaplastic thyroid carcinoma and childhood- and adolescent-onset poorly differentiated thyroid cancer (PDTC), although these tumors are rare [24, 36, 39]. Given the indolent nature of the great majority of *DICER1*-associated thyroid tumors, the SIOPE HGWG and CanGene-CanVar advise annual neck palpation from age 8 to age 20 years. In addition, patients need to be educated about possible symptoms of thyroid disease. In accordance with the recommendations of Schultz and colleagues [16], we advise thyroid ultrasound surveillance to be initiated around age 8–11 years and not to be performed more than once every three years if the baseline ultrasound does not show any suspicious nodules. Additional ultrasounds are only indicated in the case of worrisome symptoms or ultrasound findings, such as gland asymmetry or lymphadenopathy. Since interpretation of thyroid ultrasound findings in the DICER1 syndrome population is challenging, thyroid surveillance should preferably be performed in a thyroid cancer expertise center. Suspicious findings need to be discussed within a multidisciplinary team with expertise in managing patients with DICER1 syndrome.

Awareness for thyroid function monitoring is important during pregnancy in women who have undergone partial or complete thyroidectomy and in women with multinodular goiter in whom hyperthyroidism can develop [35].

### Gynecological surveillance

*DICER1*-associated gynecological tumors include ovarian sex cord-stromal tumors (especially Sertoli-Leydig cell tumor [SLCT], but also gynandroblastoma), cervical embryonal rhabdomyosarcoma (ERMS), and ovarian sarcomas [1, 3, 35].The estimated cumulative incidence of ovarian SLCT in DICER1 syndrome patients (index and non-index case patients) is ~ 7% by the age of 60 years. Gynandroblastoma, cervical ERMS, and ovarian sarcomas are rare. To date, five gynandroblastomas, fourteen cervical ERMS and three ovarian sarcomas have been reported in individuals with germline pathogenic *DICER1* variants [8, 35]. Stewart and colleagues performed Standardized Incidence Ratios (SIR) analysis of 102 nonproband *DICER1* pathogenic variant carriers [12]. They calculated that *DICER1* carriers have a markedly elevated risk of developing gynandroblastoma (SIR 1.0 × 10^5^)and SLCT (SIR 2.7 × 10^3^) compared with the expected frequencies from the SEER program.

SLCT can occur from early childhood to late adulthood, but ~ 95% of cases are diagnosed before 40 years of age, with a peak incidence between age 10 and 25 years [1, 3, 12]. Well-differentiated stage I tumors are typically treated with surgery alone, whereas higher stages require adjuvant chemotherapy [3]. Surveillance imaging might reduce higher stage presentation and the need for adjuvant therapy, although the clinical efficacy has still to be determined. Girls and women with ovarian sex cord-stromal tumors often present with symptoms of increased estrogen or androgen concentrations, including menstrual irregularity, virilization, and precocious puberty [3]. These symptoms can already be present in females with early stage tumors, triggering early diagnosis. In prepubertal girls visualization of the ovaries on transabdominal ultrasound is often challenging due to the small size of prepubertal ovaries [40]. There is no current evidence that early stage tumors can be detected with ultrasound before girls and women develop symptoms. Therefore, the SIOPE HGWG and CanGene-CanVar do not recommend standardized imaging of the ovaries but advise to primarily focus on clinical evaluation. Additional annual ultrasounds of the ovaries from age 8 to age 40 years can be considered on an individual basis and after a careful discussion of potential benefits and harms. One should not overestimate the value of an annual ultrasound to detect ovarian malignancies as it may give a false sense of security. Perspectives on surveillance can differ between individuals and between countries due to different medical cultures. In some European countries, participation in preventive health check-ups, including ultrasounds of the ovaries, is quite common in the healthy population. We recommend annual broad clinical examination from birth to age 20 years for possible signs and symptoms of *DICER1*-associated tumors. Since the peak incidence of ovarian sex-cord stromal tumors exceeds the age of 20 years, continued education on proper vigilance for symptoms of these tumors are important, especially for women who do not choose ultrasound surveillance. The information leaflet can be used for this purpose.

In the coming years, (international) data collection will hopefully provide information elucidating whether ultrasound surveillance of the ovaries reduces morbidity by facilitating earlier diagnosis and treatment of as yet asymptomatic individuals with *DICER1*-associated ovarian tumors.

### Surveillance of the eyes

CBME in individuals with DICER1 syndrome is very rare, with an estimated cumulative incidence of 3% [10]. To date, approximately ten children with CBME and pathogenic germline *DICER1* variants have been reported [10, 41–45]. The age of presentation varied between 3 and 16 years, with 90% of tumors manifesting in the first decade of life. Diagnostic delay is a common problem in patients with CBME. Most important causes of this diagnostic delay are initial misdiagnosis and mismanagement [46]. Almost half of reported CBME patients presented with secondary manifestations, like cataract, secondary glaucoma, and retrolental neoplastic cyclitic membrane [46, 47]. Some patients were treated for these conditions for a long time, or underwent surgery before recognition of the tumor [47].

Schultz and colleagues have recommended annual dilated ophthalmologic examination from 3 years of age through at least 10 years of age [16]. Dilated ophthalmologic examination may help detect CBME-associated signs, although the effect on morbidity and mortality is unclear. It is challenging to visualize a ciliary body tumor until enlargement causes secondary manifestations [46]. Therefore, it is unknown whether small, pre-symptomatic ciliary body tumors can be detected with a dilated ophthalmologic examination. In addition, dilated ophthalmologic examination can be very distressing for young children.

Given these difficulties, and the rarity of CBME in *DICER1* pathogenic variant carriers, the SIOPE HGWG and CanGene-CanVar do not recommend standardized dilated ophthalmologic examination in asymptomatic carriers. We strongly recommend education of parents and guardians about possible CBME-associated signs and symptoms since awareness of these symptoms may prevent diagnostic delay.

## Discussion

The recommendations of the SIOPE HGWG and CanGene-CanVar, summarized in a surveillance protocol and a patient information leaflet, provide a framework for the surveillance management of *DICER1* pathogenic variant carriers.

The simplified minimum surveillance program is intended to optimize the balance between benefits versus risks and burden, and to be practical. For example, equal surveillance intervals for different surveillance procedures should minimize the number of hospital visits, and pulmonary and renal surveillance both stop at age 6, which is 6–12 years earlier than recommended by others [15, 16, 37].

Besides the value and utility of a simplified surveillance program, we want to highlight the importance of patient education and shared decision making. Informing patients and families about (the limited knowledge of) age-related tumor risks, and potential benefits and harms of surveillance is fundamental to informed decision making. Studies have shown that effective information exchange between clinicians and (parents of) patients may change patients’ attitudes towards surveillance (e.g., acceptable risk threshold beliefs) and can enhance patients’ self-care skills [27, 48]. Our proposed surveillance program for consideration provides a framework for a clinician-patient discussion about potential benefits and harms of surveillance. Further research is needed to validate the clinical utility of the proposed minimum and extended surveillance program and the information leaflet.

Aside from benign thyroid disease the penetrance of germline *DICER1* pathogenic variants is generally low, but some patients or families demonstrate significantly increased penetrance [1]. For example, individuals with mosaicism for *DICER1* pathogenic missense variants in the RNase IIIb domain present with more severe *DICER1-*associated phenotypes, including higher tumor burden, earlier age of onset and greater range of phenotypes [49, 50]. To date, no other clear genotype–phenotype correlations have been identified and it is likely that genetic modifying factors contribute to phenotypic expression in carriers of a germline *DICER1* pathogenic variant [1]. Further research into the molecular characteristics of DICER1 syndrome is needed to improve our understanding of variable penetrance. Ultimately, it is hoped that patient-specific (epi)genetic factors can be taken into account when estimating *DICER1-*associated tumor risks and developing (personalized) surveillance protocols. Until then, individuals with mosaicism for *DICER1* pathogenic missense variants in the RNase IIIb domain may require more intensive surveillance then proposed in this protocol.

In the context of developing cancer predisposition syndrome surveillance protocols, fundamental decisions have to be made, such as when to start and stop surveillance. One can choose surveillance programs that cover the minimum and maximum reported age of onset of disease, or focus on the age period of highest risk—as we did—, and which is standard practice in better defined cancer predisposition syndromes such as *BRCA1/BRCA2*-associated familial breast and ovarian cancer and Lynch syndrome. There is a fine balance between how much surveillance is “too much” versus “not enough” and we are aware that perspectives on surveillance can differ between countries due to different (medical) cultures and health insurance systems [51, 52]. Most European countries have a universal health care system, based on solidarity, equality and social responsibility, and funded through taxes (by public authorities) and/or social contributions (by employers and employees). In this context, critical evaluation of benefits and harms of surveillance is essential to justify the efforts and costs of surveillance. Although some may prefer more extensive surveillance programs, we feel that our simplified DICER1 syndrome surveillance protocol best reflects European perspectives on the impact of surveillance on daily life and its costs.

Progress has been made in the awareness of DICER1 syndrome, the associated tumors and ages of onset, although, as mentioned previously, many important research questions remain. Directions for future research are the natural history and growth rate of *DICER1*-associated tumors (especially lung cysts and thyroid nodules), the prevalence of adult tumors associated with DICER1 syndrome, and the clinical utility of proposed DICER1 syndrome surveillance protocols. Prospective data are needed to validate the clinical utility of proposed DICER1 syndrome surveillance protocols [15, 16, 37, 52]. The SIOPE HGWG and CanGene-CanVar strongly encourage enrollment of patients in the International Pleuropulmonary Blastoma (PPB)/DICER1 Registry (www.ppbregistry.org) to expand the evidence base for surveillance recommendations and to refine DICER1 syndrome penetrance. In Europe, research procedures must consider General Data Protection Regulation (GDPR) compliance. Patients may consider individual registration and/or national *DICER1* registries can feed anonymized data into the International PPB Registry where it could be analyzed and used to monitor the efficacy and patient tolerance of proposed surveillance protocols. To ensure ongoing optimization, we will reevaluate the proposed surveillance recommendations as new information becomes available.

## Supplementary Information

Below is the link to the electronic supplementary material.

Supplementary file1 (DOCX 14 kb)
