# Supplementary material for: Surveillance recommendations for DICER1 pathogenic variant carriers: a report from the SIOPE Host Genome Working Group and CanGene-CanVar Clinical Guideline Working Group
Source: Fam Cancer. 2021 Jun 25;20(4):337–48. doi: 10.1007/s10689-021-00264-y (PMC8484187; doi:10.1007/s10689-021-00264-y)
Supplement: Supplementary file 1 — Supplementary file1 (DOCX 14 kb) [file 10689_2021_264_MOESM1_ESM.docx]

**Supplemental table 1.** Search terms used to identify relevant studies on currently known *DICER1*-associated tumors and surveillance.

| **Topic** | **Search terms: “DICER1”[tiab] AND “humans”[Mesh]** |
| --- | --- |
| **General** | (“surveillance”[tiab] OR “screening”[tiab]) OR “incidence”[tiab] OR “penetrance”[tiab]) |
| **Pulmonary tumors** | (pleuropulmonary blastoma”[tiab] OR “PPB”[tiab] OR “lung cyst”[tiab]) |
| **Renal tumors** | (“cystic nephroma”[tiab] OR “CN”[tiab] OR Wilm*[tiab] OR nephroblastoma*[tiab] OR “renal sarcoma”[tiab] OR “sarcoma of kidney”[tiab] |
| **Thyroid tumors** | (“multinodular goiter”[tiab] OR “MNG”[tiab] OR “thyroid”[tiab]) OR “Thyroid Neoplasms”[Mesh] |
| **Gynaecological tumors** | “ovarian”[tiab] OR “sex cord stromal tumor”[tiab] OR “sertoli leydig cell tumor”[tiab] OR “SLCT”[tiab] OR “gynandroblastoma”[tiab] OR “embryonal rhabdomyosarcoma”[tiab] OR “ERMS” OR “gynecologic“[tiab] OR “Ovarian Neoplasms”[Mesh] OR “Sex Cord-Gonadal Stromal Tumors”[Mesh] |
| **Eye tumors** | "ciliary body medulloepithelioma"[tiab] OR “CBME”[tiab] OR “ocular medulloepithelioma”[tiab]) |
| **Other tumors** | “hamartoma”[tiab] OR “sarcoma”[tiab] |
